# Cellular Plasticity of Mammary Epithelial Cells Underlies Heterogeneity of Breast Cancer

**DOI:** 10.3390/biomedicines6040103

**Published:** 2018-11-01

**Authors:** Verónica Rodilla, Silvia Fre

**Affiliations:** 1 Preclinical Research Program, Vall d’Hebron Institute of Oncology, 08035 Barcelona, Spain; 2 Institut Curie, PSL Research University, INSERM U934, CNRS UMR3215, F-75248 Paris CEDEX 05, France

**Keywords:** lineage tracing, stem cells, multipotency, cellular plasticity, mammary gland, breast cancer

## Abstract

The hierarchical relationships between stem cells, lineage-committed progenitors, and differentiated cells remain unclear in several tissues, due to a high degree of cell plasticity, allowing cells to switch between different cell states. The mouse mammary gland, similarly to other tissues such as the prostate, the sweat gland, and the respiratory tract airways, consists of an epithelium exclusively maintained by unipotent progenitors throughout adulthood. Such unipotent progenitors, however, retain a remarkable cellular plasticity, as they can revert to multipotency during epithelial regeneration as well as upon oncogene activation. Here, we revise the current knowledge on mammary cell hierarchies in light of the most recent lineage tracing studies performed in the mammary gland and highlight how stem cell differentiation or reversion to multipotency are at the base of tumor development and progression. In addition, we will discuss the current knowledge about the interplay between tumor cells of origin and defined genetic mutations, leading to different tumor types, and its implications in choosing specific therapeutic protocols for breast cancer patients.

## 1. Introduction

Lineage tracing is now considered the most reliable approach to study cellular hierarchies and cell fate in vivo. This type of clonal analysis consists of using a genetic (hence heritable) label to target specific cells and follow their destiny and progeny in vivo [1]. The most commonly used lineage tracing approach uses an inducible form of the Cre recombinase to trigger the permanent expression of a reporter gene in defined cells. This strategy has been used to study cell fate and evaluate stem cell potency in vivo in most tissues. Such an approach has often been employed to define novel stem cell markers, such as Lgr5 in the small intestine [2], as well as to discover the existence of unipotent stem cells in the mammary gland, prostate, or lung [3,4,5,6].

In some organs, like the intestine or the brain, clonal analyses have proved the existence of multipotent adult stem cells able to give rise to all differentiated cell types of their tissue of origin [7,8]. In the mammary gland, instead, the hierarchical relationships between stem cells and progenitors are less clear. Recent lineage tracing approaches, predominantly performed using specific cytokeratin promoters, have indeed given conflicting results, and the existence of adult multipotent stem cells in this tissue is still under debate. Interestingly, it has been shown that under different stress conditions, such as transplantation, enzymatic digestion, or oncogene expression, cells can undergo lineage conversion, changing their physiological potency and commitment [3,9,10,11,12]. These observations have important implications for understanding the molecular mechanisms underlying cellular plasticity and fate alteration, a topic with profound implications for the development of novel cancer therapies.

The mammary ductal tree is composed of two epithelial compartments: polarized cells facing the ductal lumen are called luminal cells (LCs), and cells found in the outer layer, in contact with the basement membrane, are myoepithelial cells with the ability to contract thanks to the expression of smooth muscle actin (SMA), generally termed basal cells (BCs). Luminal cells can be further subdivided into two independent subpopulations, based on the expression of the hormone receptors estrogen alpha (ERα) and progesterone (PR) [13]: ERα-positive (hormone sensing) and ERα-negative (hormone responding) cells. The mammary gland is highly sensitive to the paracrine effects of the steroid hormones estrogen and progesterone, particularly during pregnancy when ERα-negative cells rapidly expand forming milk-producing alveoli [14].

## 2. Lineage Tracing Studies in the Mouse Mammary Gland

Pioneering studies that explored the capacity of single mammary cells to reconstitute a whole functional gland when orthotopically transplanted in the cleared fat pad of host mice revealed that a small subset of BCs is extremely efficient in mammary reconstitution experiments, even at very low density, establishing that the basal mammary compartment harbors multipotent mammary stem cells (MaSCs), initially believed to be responsible for homeostatic tissue maintenance throughout adult life [15,16]. However, it has later been demonstrated that, notwithstanding the much higher transplantation efficiency of BCs, specific subsets of LCs can generate outgrowths in mammary reconstitution assays [3,17,18], revealing that this assay measures cell plasticity and not in vivo stem cell potency. In other words, mammary transplantation assays can tell us what a specific cell can do, but not necessarily what it actually does in homeostatic conditions.

**Figure 1:**
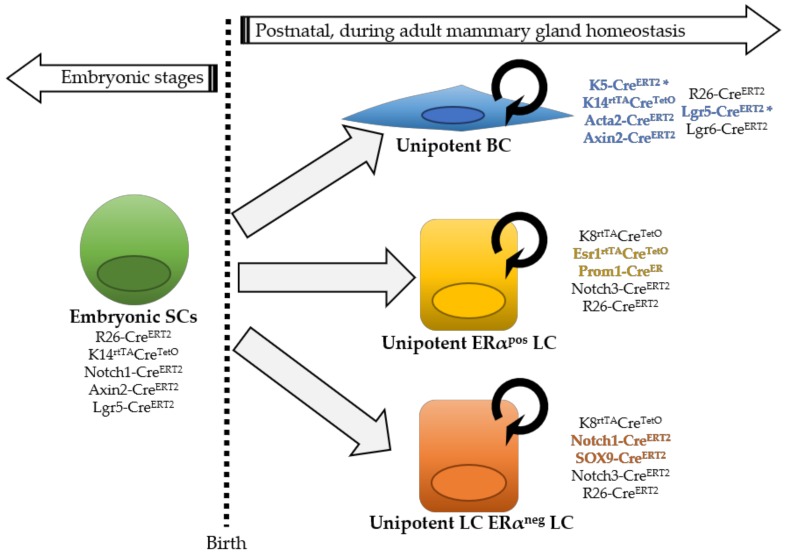
Model of mammary epithelial cell hierarchy based on lineage tracing studies. Multipotent stem cells (SCs) are found exclusively during embryonic development, while after birth distinct unipotent progenitors are responsible for sustaining tissue growth and homeostasis, giving rise to each mammary cell type: basal cell (BC), estrogen alpha (ERα)-positive luminal cell (LC) and ERα-negative LC. The asterisk in K5-Cre^ERT2^ and Lgr5-Cre^ERT2^ indicates that, depending on different mouse lines, cell targeting might be exclusively basal or also include some rare LCs. Next to each cell type, the different inducible Cre lines that have been used to target them are indicated. Mouse lines that exclusively label one epithelial cell type are colored.

Initial lineage tracing studies using luminal (K8) or basal cytokeratin (K5, K14) promoters indicated that in the adult homeostatic mouse mammary gland, BCs and LCs could only generate BCs and LCs, respectively, and suggested that both epithelial compartments were maintained independently by two different populations of unipotent stem cells [3] (Figure 1). However, another study evoked the existence of rare BCs that were found capable of producing both a basal and luminal progeny in adult mice, suggesting multipotency [19]. Part of these discrepancies is explained by the use of different regions of the K5 promoter (BC marker), changing its cell-specificity; in fact, the lack of short time points, demonstrating that the K5-Cre^ERT2^ mouse line used in the study by *Rios* et al. targeted exclusively BCs, represents a main criticism, preventing substantiated conclusions from being drawn. These conflicting results have been clarified more recently through the use of clonal analysis at saturation, allowing the assessment of the fate of all cells of a given compartment (BCs with K14^rtTA^-Cre^TetO^ and LCs with K8^rtTA^-Cre^TetO^), resulting in a definitive demonstration of a lack of multipotent stem cells in the postnatal mouse mammary gland [20]. It should be noted that, as lineage tracing approaches are not feasible in the human context, some differences in the cellular hierarchy might exist between the mouse mammary gland and the human breast.

Instead of using cytokeratin promoters, targeting in a rather general way all cells in a given epithelial compartment, other groups have approached this question by genetically marking specific cells with different promoters, as illustrated in Figure 1: Axin2-Cre^ERT2^, marking Wnt/β-catenin-responsive cells throughout mammary gland development [21]; αSMA (Acta2-Cre^ERT2^ [22]) targeting exclusively postnatal myoepithelial cells, similarly to K5 or K14. Clonal analysis using Dll1-Cre^ERT2^, Lgr5-Cre^ERT2^ or Lgr6-Cre^ERT2^ lines could not reach a definitive consensus on the existence of unipotent or multipotent MaSCs, as these genes are predominantly expressed in BCs, but also in some LCs [3,23,24,25]. Rosa26-Cre^ERT2^ mice, using a ubiquitous promoter, have instead been used to achieve unbiased labeling of single proliferating cells [26,27].

Moreover, the promoters of different Notch receptors, SOX9, PROM-1, and ERα, have been used to gain insights into the cellular hierarchy within the luminal compartment. Unlike *Notch3,* labeling both ERα-positive and ERα-negative LCs, the *Esr1* and *Prom1* genes mark exclusively ERα-positive LCs, whereas *Notch1* and *Sox9* target uniquely ERα-negative LCs in the postnatal gland [17,28,29,30]. Collectively, all these studies provided strong evidence that in adult mice, BCs and LCs are entirely self-sustained by unipotent progenitors, and this holds true for ERα-positive and negative luminal subsets, representing two independent lineages. Indeed, all these cell populations sustain their respective lineage throughout adulthood, even after serial pregnancies, demonstrating long-term self-renewal capacity (Figure 1).

## 3. Mammary Gland Development

The development of the mammary gland is a multistage process, starting during embryogenesis and terminating at the end of puberty. In mice, embryonic mammogenesis initiates around E11.5, when the ectoderm invaginates to form a mammary placode, which will form the mammary bud [31,32]. The nipple is formed from epidermal cells overlying the bud, and a lumen is formed in the first rudimentary duct at E16.5. Mammary development involves cell proliferation and elongation of the initial sprout, arising around E15.5, which will give rise, at birth, to a rudimentary ductal tree [31]. Under strong hormonal influence at puberty, the ductal epithelium undergoes extensive remodeling involving ramification and elongation within the mammary fat pad in a process referred to as branching morphogenesis [33]. The terminal end buds (TEBs) are highly proliferative structures formed at the tip of growing ducts, which contain an outer layer of cap cells surrounding multilayered highly proliferating epithelial cells (body cells) [31]. Even after branching morphogenesis completes at the end of puberty, the mammary epithelium undergoes tremendous transformation during each pregnancy and lactation, when it becomes a milk-producing organ, and at weaning, when the gland involutes to revert to a resting state [34].

**Figure 2:**
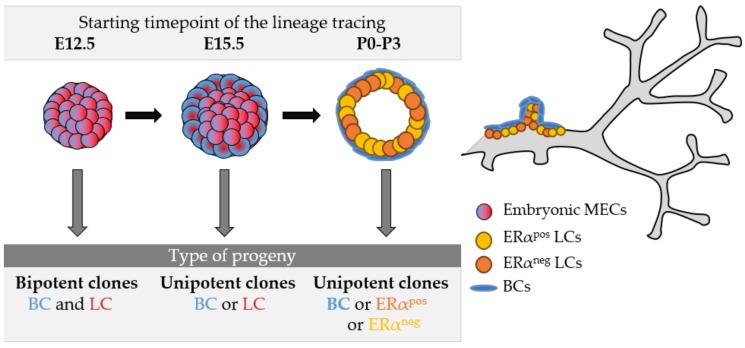
Model of mammary gland development based on lineage tracing studies. At E12.5, a pool of mammary cells that co-expresses basal and luminal markers presents multipotent capacity. At 15.5, the mammary bud contains only lineage-restricted BCs or LCs, regardless of their co-expression of basal and luminal markers. After birth, the three mammary epithelial lineages are completely specified and self-maintained. The type of progeny observed depending on the developmental stage of clonal analysis is shown in the grey table. MECs, mammary epithelial cells.

Although it has been shown that embryonic MaSCs express a hybrid signature comprising markers of both luminal and basal cell types [17,35], it remained unclear when and how multipotent progenitors make the switch to unipotency. Indeed, previous population-based studies lacked the resolution required to address the important question of whether individual embryonic stem cells exhibit multipotent potential at the clonal level or if they instead comprise distinct stem cell subsets committed toward a specific cell lineage. To determine whether embryonic mammary cells exhibit bipotency at the cellular level and, if so, when this potential is lost during development, a recent study has performed multicolor clonal analysis at different embryonic and perinatal times, identifying the critical developmental times for cell identity acquisition for all mammary cell types [10]. Supported by mathematical modelling and statistical analysis of the observed clonal dynamics, the results obtained indicated that early embryonic development of the mouse mammary gland relies on the proliferative activity of multipotent MaSCs that progressively differentiate into lineage-restricted unipotent precursors that fuel late embryonic and post-natal growth. Unexpectedly, this in vivo study revealed that mouse embryonic mammary glands contain exclusively unipotent lineage-committed stem cells from day E15.5, surprisingly early in mammogenesis (Figure 2).

## 4. Cellular Plasticity in the Mammary Gland

Collectively, the evidence derived from lineage tracing analyses indicates that multipotent MaSCs exclusively exist during embryonic mammary gland development. Notwithstanding their early lineage commitment though, mammary cells retain an extraordinary plasticity upon ectopic expression of key proteins or following transplantation [3,17].

Several functional studies have contributed to our understanding of stem cell plasticity in vivo. Some crucial factors have been shown to induce lineage conversion in the mammary epithelium, such as Slug or p63, strong basal determinants. Perturbation of either of these genes can alter mammary homeostasis; for instance, upon *Snai2*/Slug knockdown, the expression of several luminal markers was aberrantly induced in mammary epithelial cells (MECs) [36]. The basal marker p63 has been described as a master regulator of basal fate specification, since the overexpression of *Trp63* in committed luminal cells was sufficient to induce basal differentiation of the targeted cells, while its downregulation would favor a luminal fate [9,37].

On the other hand, Notch1 was found to have an opposing role, pushing MECs to acquire a luminal identity, specifically ERα-negative LCs [10,11,37]. Gata-3 is a crucial regulator of the luminal lineage and is sufficient to induce milk protein gene expression in BCs even in the absence of a lactogenic stimulus [38]. C/EBPb also specifies luminal cell fate in the mammary gland, and its loss induces misexpression of basal markers in the luminal cell compartment [39].

Additionally, forced expression of lineage determinants can impose a specific terminal cell fate on multipotent embryonic mammary stem cells: Notch1 activation or p63 expression instructs embryonic cells to give rise to a unique cell lineage, LC ER^neg^ or BC, respectively [9,10].

The expression of intrinsic cell fate determinants is likely dependent on signals from surrounding cells, and is therefore linked to the position that each cell acquires during mammary branching morphogenesis. In colon cancer, for example, a recent study elegantly showed that the microenvironment is dominant over cell-autonomous features in defining stem cell functionality [40]. Likewise, in mammary reconstitution assays, the fact that sorted BCs and LCs maintain their unipotent lineage commitment when co-transplanted, but they reacquire multipotency when isolated BCs or LCs are grafted in an empty fat pad, exemplifies the dramatic influence of the cell microenvironment on cell plasticity [3].

Together, all these studies have proven the extensive plasticity of mammary cells, which can switch cell fate upon misexpression of key determinants. Such lineage conversion possibly involves reactivation of multipotency programs, carrying profound implications for cancer development.

## 5. Breast Cancer

Breast cancer is divided into five subtypes based on their transcriptional profiles: luminal A, luminal B, HER2-positive, claudin-low, and basal-like [41]. Comparative gene expression signatures established an association between different mammary epithelial populations and specific breast cancer subtypes; for example, a BCs signature was closely aligned with the claudin-low subtype, while the ERα-negative profile was closer to the basal-like subtype, and the ERα-positive signature correlated with the luminal A tumor type [41]. It remains unclear, however, why the other subtypes (luminal B and HER2-positive) were not aligned to any particular MEC population. This type of comparative analysis suggested that the cell of origin of a tumor might be critical to generate a distinct subtype of breast cancer.

Several recent studies have revealed that LCs represent the “cells of origin” of basal-like breast cancer. Indeed, generation of basal-like tumors was predominantly observed upon either BRCA1 knockdown or PI3K activation in LCs rather than in BCs [42,43,44]. Using elegant genetic marking approaches in mice, two independent groups showed that expression of oncogenic PI3K in adult BCs induced the formation of luminal ERα-positive tumors, while its expression in LCs produced both luminal and basal-like tumors, with a considerably shorter latency [42,43]. Additionally, it has been demonstrated that polyoma middle T (PyMT) antigen or ErbB2 signaling activation in LCs can reprogram cells to multipotency, making them able to give rise to BCs [45]. Moreover, other studies have associated epigenetic reprogramming, hypoxia, and DNA damage, all stimuli that have been linked to breast cancer, with MECs dedifferentiation [46,47,48]. Those studies suggested that adult cells undergo reprogramming to a multipotent stem cell state during tumorigenesis, supporting the concept that cancers may arise from reactivation of embryonic developmental programs in postnatal tissues.

So, if the expression of some oncogenes or the loss of tumor suppressors can switch the cell fate of MECs, then what becomes really critical, rather than the cell of origin, is the genetic programs that are deregulated in these cells. A large number of genome-wide sequencing studies of breast cancer established that common mutations are characteristics of specific breast cancer subtypes [49]: while mutations in the tumor suppressor p53 and activating mutations in PI3K are commonly found across different tumor subtypes, Rb is frequently associated with luminal B and basal-like tumors [50]. All these studies suggest the need to redefine the subtypes of breast cancer in order to improve the therapies of choice for women diagnosed with breast cancer.

Nowadays, therapies used to treat breast cancer depend on the clinical subtype diagnosed. These subtypes are defined based on the histological expression of three markers: ERα, HER2, and the proliferation marker Ki67. Patients with ERα-positive tumors termed as luminal A or B, depending on the percentage of Ki67, receive hormone therapies (tamoxifen or fulvestrant); HER2-positive tumors are treated with HER2 targeted therapies, such as monoclonal antibodies (trastuzumab or pertuzumab), antibody-drug conjugates (i.e., T-DM1), or tyrosine kinase inhibitors (i.e., lapatinib). For tumors lacking expression of ERα, PR and HER2, classified as triple-negative breast cancer (TNBC), considered the most aggressive subtype and currently lacking targeted therapies, the protocol recommends the choice of anthracyclines or taxanes, which non-specifically target highly proliferating cells [41].

## 6. Conclusions and Future Perspectives

As discussed above, several lineage tracing studies have now proved that during mouse mammary gland development, multipotent MaSCs are replaced by unipotent progenitors as early as E15.5, and that the adult mammary gland is sustained by unipotent stem cells with long-term self-renewal capacity.

The studies performed up to now, however, carry the intrinsic flaw of not labelling all mammary cells at a given developmental stage, so the existence of rare multipotent cells that were not targeted by those approaches cannot be completely excluded. Only clonal analyses at saturation in embryos, similar to the one performed in adult glands by Blanpain and colleagues [20], could ensure that no cell is missed. In addition, unbiased barcoding approaches to systematically and quantitatively define the amount of multipotent and lineage-committed MaSCs in the embryonic gland should be used, allowing the marking of all cells individually with unique heritable barcodes.

Altogether, it is becoming clear that unipotency of MaSCs belies a remarkable degree of plasticity that allows cell autonomous factors to redirect cell identity and differentiation potential, irrespective of the degree of commitment of the targeted cells. Understanding the mechanisms allowing reactivation of multipotency programs in lineage-committed mammary progenitors should help decipher the signals underlying cell identity acquisition and plasticity in the developing mouse mammary gland and how different cellular states relate to oncogenesis, as well as to the broadly documented intratumoral heterogeneity of breast cancer.

If we assume that, as in mice, the human breast is maintained by lineage-restricted cells, the heterogeneity found in tumors could be explained by the reactivation of multipotent embryonic program during tumorigenesis. As mentioned above, in mice it has been shown that PI3K signaling activation is sufficient to generate multipotent MaSCs that can give rise to both LCs and BCs, even before forming tumors [42]. Indeed, it has been observed that some embryonic mammary signatures, when re-expressed in adult cells, lead to tumor formation [35,51].

Given the extensive intratumoral heterogeneity, the fact that the choice of treatment depends exclusively on the expression of three markers with a very heterogenous pattern within the same tumor is worrisome, and it is not surprising that some patients do not respond to therapies or suffer from relapse. This is particularly critical in patients diagnosed with TNBC, since treatments, when available, do not consider the cellular identity of the tumor cells. Our knowledge on mammary gland hierarchies, the relevant factors that determine cell fate of specific cells, and the molecular signatures of different mammary cell types should help in defining combined targeted therapies that could eliminate specific tumor cell subsets. However, therapies specifically targeting the “cancer stem cell” fraction of a tumor, as defined by specific markers, are likely to fail because unipotent or differentiated cells display tremendous plasticity and can become clonogenic and contribute to tumor growth when they gain access to the right niche, as has been recently shown in colon cancer [52,53].
